# Cognitive Rehabilitation in Bilateral Vestibular Patients: A Computational Perspective

**DOI:** 10.3389/fneur.2018.00286

**Published:** 2018-04-27

**Authors:** Andrew W. Ellis, Corina G. Schöne, Dominique Vibert, Marco D. Caversaccio, Fred W. Mast

**Affiliations:** ^1^Department of Psychology, University of Bern, Bern, Switzerland; ^2^Center for Cognition, Learning and Memory, University of Bern, Bern, Switzerland; ^3^Department of Otorhinolaryngology, Head and Neck Surgery, Inselspital, University Hospital Bern, University of Bern, Bern, Switzerland

**Keywords:** cognitive training, bilateral vestibulopathy, bilateral vestibular loss, rehabilitation, vestibular cognition, computational modeling, mental imagery, self-motion perception

## Abstract

There is evidence that vestibular sensory processing affects, and is affected by, higher cognitive processes. This is highly relevant from a clinical perspective, where there is evidence for cognitive impairments in patients with peripheral vestibular deficits. The vestibular system performs complex probabilistic computations, and we claim that understanding these is important for investigating interactions between vestibular processing and cognition. Furthermore, this will aid our understanding of patients’ self-motion perception and will provide useful information for clinical interventions. We propose that cognitive training is a promising way to alleviate the debilitating symptoms of patients with complete bilateral vestibular loss (BVP), who often fail to show improvement when relying solely on conventional treatment methods. We present a probabilistic model capable of processing vestibular sensory data during both passive and active self-motion. Crucially, in our model, knowledge from multiple sources, including higher-level cognition, can be used to predict head motion. This is the entry point for cognitive interventions. Despite the loss of sensory input, the processing circuitry in BVP patients is still intact, and they can still perceive self-motion when the movement is self-generated. We provide computer simulations illustrating self-motion perception of BVP patients. Cognitive training may lead to more accurate and confident predictions, which result in decreased weighting of sensory input, and thus improved self-motion perception. Using our model, we show the possible impact of cognitive interventions to help vestibular rehabilitation in patients with BVP.

## Introduction

Patients with bilateral vestibulopathy suffer from a severely reduced (incomplete vestibular loss) or totally absent function (complete vestibular loss) of both vestibular end organs, vestibular nerves, or a combination thereof (1). The main symptoms of BVP are unsteadiness of gait, oscillopsia, and postural imbalance (particularly pronounced in darkness) with more pronounced symptoms and worse course of disease in BVP patients with complete vestibular loss. These symptoms are directly related to dysfunctional perception of self-motion (2, 3). There is evidence that vestibular information is nested and intertwined with higher cognitive processes [see Ref. (4) for a review]. Accordingly, there is growing evidence for cognitive impairments in vestibular patients. Deficits in visuospatial abilities such as mental rotation, spatial navigation, or spatial memory have been shown in patients with BVP (5–7), indicating a changed internal spatial representation (8). Furthermore, atrophy of the hippocampus is strongly correlated with impaired visuospatial abilities in patients with BVP (5, 9). BVP can also lead to cognitive impairments in non-spatial cognitive domains, such as problems with concentration, short-term memory, reading abilities, or executive functions (5, 10–12).

Conventional treatments for patients with BVP include counseling and daily intensive vestibular physical therapy, in combination with neurotological rehabilitation. The goals of vestibular rehabilitation therapy are to improve gaze and postural stability, minimize falls, decrease the sense of disequilibrium, and prevent an increasingly sedentary lifestyle (13). Traditional vestibular rehabilitation therapy includes exercises to promote alternative strategies for gaze stability by potentiation of the cervico-ocular reflex, modification of saccades (decreasing the amplitude of saccades/corrective saccades), increasing smooth pursuit eye movement or central pre-programming of eye movements [for a review, see Ref. (14)]. Self-motion perception can be induced by exposure to large-field visual stimuli (optokinetic stimulation) with the goal of developing compensation by increasing the weight of visual cues. Recent literature suggests using covert saccades during head movements as rehabilitation strategy in patients with BVP, since elimination of oscillopsia was observed in BVP patients who made covert saccades (15, 16). Even though conventional therapies are applied to patients with BVP, recovery is usually incomplete (1).

A largely unexplored approach to rehabilitation is cognitive training (17). The authors suggest that cognitive training methods can lead to reduced symptoms and improved compensation for the lack of sensory signals in patients with complete vestibular loss. While cognitive training has not been applied to BVP patients, its utility for improving balancing ability has been shown in elderly people and people with mild cognitive impairment or dementia (18, 19). The link between cognition and balancing abilities is further shown in cognitively impaired patient groups (20–22). Cognitive training may provide many benefits; it is cost-effective, and can easily be performed on a daily basis in the comfort of patients’ own homes. Additionally, patients do not depend on medication and can take action to reduce their symptoms, which enhances their self-efficacy. As described above, vestibular deafferentation has dramatic consequences for higher-order processing of vestibular and spatial information, and cognitive training might be a promising opportunity for treating the adverse consequences of BVP. Conventional treatment attempts to reduce the reliance on abnormal vestibular sensory signals and improve the use of non-vestibular sensory signals. Cognitive training operates at a higher level of processing. In this paper, we explore how cognitive training might aid rehabilitation of BVP patients by considering the computations involved in vestibular processing, the complexity of which is often underestimated.

## A Computational Model of Sensory Inference

It seems impossible for BVP patients to extract any kind of information about their head movements, because their vestibular signals provide little or no information. However, sensory processing involves much more than extracting information from noisy and ambiguous sensory data; sensory information is combined with prior knowledge about the world (23). Many tasks, from perception (24, 25) to higher-level cognition (26), have been described in a Bayesian framework. Additionally, when timing is essential, as is the case in the vestibular system, purely data-driven processing would lead to time lags. Instead, the brain continuously makes predictions and uses the sensory data to correct those predictions. Decades of vestibular research have provided insight into the type of computations used by the brain (27–30). These can be described as filtering algorithms, which rely on probabilistic models of the dynamics of head movements and the sensory data. The dynamics of head movements (e.g., the head velocity) are represented as a latent process (process model), and the sensory data are represented as depending on this latent process (sensor model). The process model represents the brain’s knowledge about the laws of physics, whereas the sensor model represents the brain’s knowledge about sensor characteristics.

**Figure 1:**
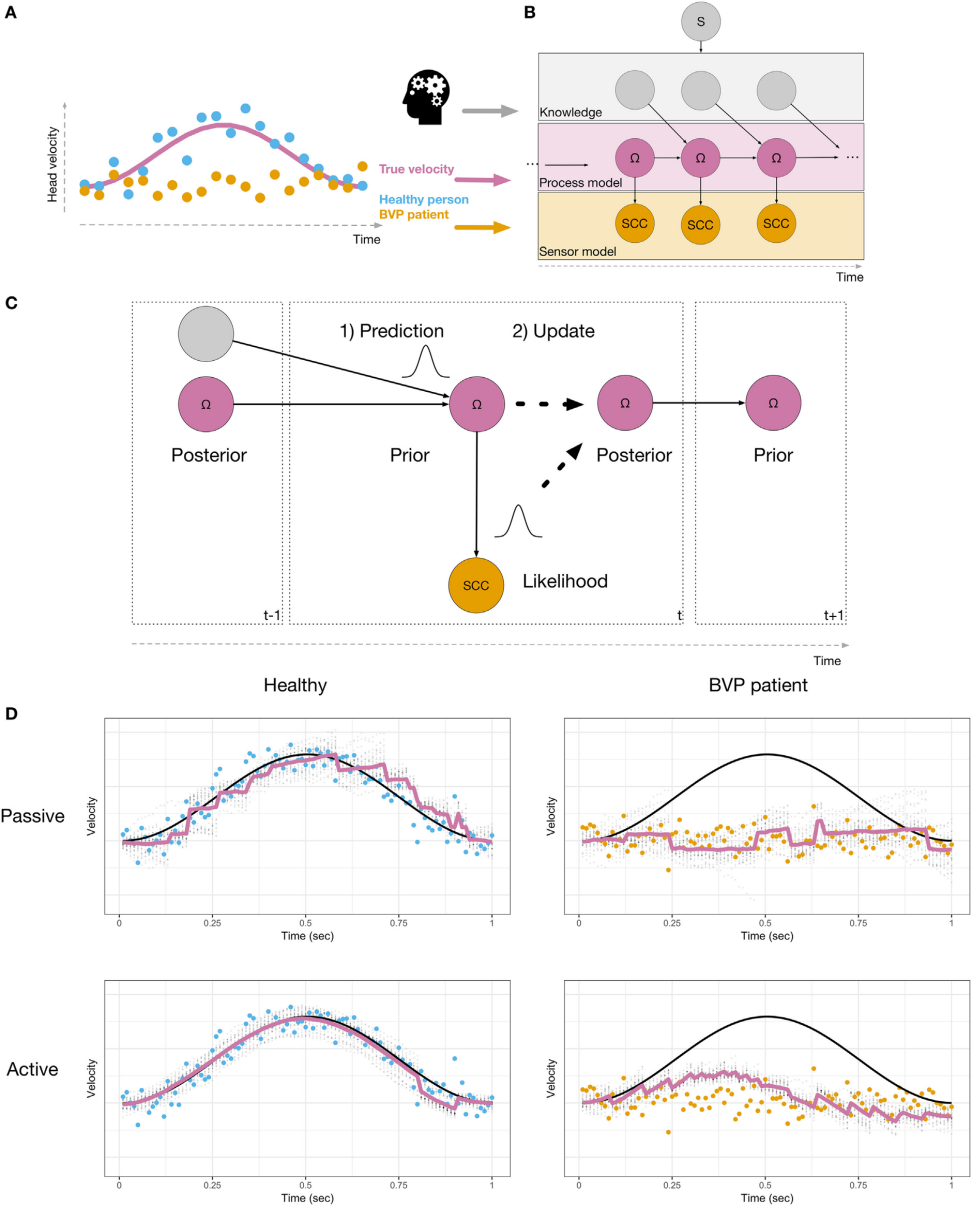
**(A)** Head velocity, shown as a pink line, during a left head turn. Sensory signals are shown in blue for a healthy person, and in orange for a BVP patient. **(B)** Probabilistic model used to perform sensory inference. The velocity Ω (pink nodes) is represented as a sequence of latent states, which need to be inferred, given the sensory observations (orange nodes). Additional knowledge (gray nodes, e.g., derived from motor commands or higher-level knowledge) are used in order to predict Ω. The model includes a binary switch S (gray node), which indicates whether or not knowledge is used (active or passive). **(C)** During inference, the current state is probabilistically predicted (1), based on the posterior from the previous time step and additional knowledge. The prediction is then updated with the likelihood (2), resulting in a posterior state estimate. This is repeated at every time step. **(D)** Simulations of a healthy person (left) and a BVP patient (right) using a particle filtering algorithm and the generative model shown in **(B)**. True velocity is shown in black. Sensory observations are shown as blue (healthy) or orange (BVP patient) dots. Estimated velocity is shown as a pink line, along with the uncertainty in the estimate (gray dots).

We present the computational principles using a simplified model of a rotation of the head about the earth-vertical (yaw) axis (Figure 1A, angular velocity as pink line). This velocity has to be inferred, using the sensory signals provided by the semicircular canals (SCC). The sensory signals of a healthy person (blue dots) are measurements of the true velocity, with added noise. In contrast, the sensory signals of a BVP patient (orange) cannot track the head velocity. Instead, they merely reflect neuronal noise. The velocity can result from either a passive movement, or an actively initiated movement. In both cases, the head velocity and the resulting sensory signals are identical (31). If the movement was self-initiated, the brain has information about the motor commands (e.g., an efference copy), which is used by the brain in order to attenuate neurons in the vestibular nuclei (32). Recently, Laurens and Angelaki (33) demonstrated that the probabilistic model used to process passive movements also applies when movements are self-initiated. Information about expected head motion must be translated into expected sensory signals, in order to compute prediction errors. The computations involved can be described as a probabilistic graphical model (Figure 1B). Head velocity is represented by state variables (Ω) evolving over time according to a process model, which represents knowledge about the physical laws of head movements. If the movement was actively generated, knowledge from the motor commands is used to compute the next state. The sensor model describes how the noisy SCC measurements arise, given the state of the head. This type of probabilistic graphical model can be used for various tasks (34). For example, imagined movement may correspond to running this model in an off-line simulation mode (35–37). In order to infer head velocity, a filtering algorithm performs sequential Bayesian inference, i.e., the brain combines prior knowledge with sensory data to obtain a posterior estimate. This is illustrated in Figure 1C. First, during the prediction step (1), a prior is created by predicting the head velocity Ω for the current time t, based on its past estimate. This prediction is probabilistic; the width of the distribution reflects how certain the brain is in its prediction. Second, in the update step (2), the sensory measurement (likelihood) is used to update the prior, resulting in a posterior estimate of Ω. If the head movement was the result of an intended action, then, knowledge about the head movement (e.g., efference copy) may be used in order to make more precise prior predictions for the state Ω. A few recent studies demonstrate that the brain must be able to use not only knowledge derived from motor commands, but also from other sources; vestibular signals can be predicted when these do not result from active self-motion (38). The brain is able to construct models of head dynamics based on various sources of knowledge. This information may be derived from other sensory modalities, such as vision or proprioception, from memory of recent movements (38) or prior knowledge obtained by verbal instruction (39). A recent study demonstrated that higher-level prior knowledge plays an important role in self-motion-related perception and decision-making (40). Thus, prior knowledge in different forms can affect vestibular perception. Mertz et al. (41) showed that imagined self-motion either facilitates or impedes the ability to detect linear accelerations, depending on the compatibility of the directions of imagined and actual motion. Nigmatullina et al. (42) found effects of imagined self-motion at the earliest stages of vestibular processing; the onset of the vestibulo-ocular reflex (VOR) was shortened when participants imagined moving in the same direction as the subsequent actual motion. Participants’ perception of self-motion was affected in a similar manner. Finally, there is evidence that the gain of the VOR depends on the distance of an imagined target (43). All of these findings point to the fact that vestibular processing must contain a great deal of flexibility and cognitive penetrability (44, 45), in the sense that information that is not directly sensorimotor in nature may affect sensory inference.

The fact that vestibular sensory processing involves a high degree of flexibility creates new possibilities for rehabilitation.

We illustrate this idea using a particle filtering algorithm (46, 47) to simulate a healthy person and a BVP patient inferring their head velocity Ω during a 1-s leftward head turn. A particle filter recursively performs a sequence of computations. First, the state is predicted, according to a model of the process being estimated. This predicted state serves as the dynamic prior. Second, an observation is used to update the prior, resulting in a posterior estimate. The amount of updating that occurs depends on how well the sensory data were predicted, given the predicted state. Figure 1D shows the results of our simulations. The top row shows passive motion and the bottom row shows an active, self-initiated movement. The healthy person is able to accurately infer the velocity in both conditions. During passive motion, when the trajectory cannot be predicted, the algorithm requires the sensory data in order to update its 1-step ahead predictions during online sensory inference. This means that the estimated velocity lags the actual velocity. During the self-initiated movement, additional knowledge results in predictions that follow the actual velocity more closely, and the lag is reduced when compared to passive motion. The situation is dramatically different for the BVP patient. During passive movement, the dynamic prior does not follow the actual velocity. In order for inference to be accurate, the brain must update the prior using the sensory signals. In BVP patients, the sensory signals do not provide information about the actual velocity. Thus, the resulting velocity estimate remains at zero—the BVP patient is unable to detect self-motion by means of vestibular information. The patient is only able to construct a better prior estimate of the velocity when the trajectory of motion is predictable. Thus, when self-motion is self-initiated, the patient perceives a velocity in the correct direction, but with decreased amplitude, compared to the true velocity. The resulting posterior estimate is drawn toward the data. This also qualitatively captures the fact that BVP patients show VOR with reduced gain (48, 49).

## Implications for BVP Patients

The perception of self-motion relies on estimates obtained from multiple sensory modalities. The goal of most conventional rehabilitation methods is to enable BVP patients to increase the weighting of visual and somatosensory cues. In the context of the computational model of the vestibular system, this can be interpreted as enabling patients to rely less on sensory signals provided by the vestibular sensors and more strongly on their dynamic prior while performing Bayesian inference. However, this requires the use of some kind of knowledge to predict the velocity. The simulations illustrate the fact that BVP patients are unable to infer their head velocity during passive movements, due to the chronic loss of sensory input. If a probabilistic model with additional knowledge can be used because the movement is predictable, inference about head velocity can be substantially improved. In addition to this, the mismatch between expected and actual sensory signals needs to be given less weight. While the reweighting of sensory signals may require long-term learning and adaptation, the use of prior knowledge is more flexible. Thus, we claim that cognitive training, if it can be shown to be effective in BVP patients, should operate by enabling patients to improve vestibular sensory inference through the use of prior knowledge during sensory inference.

## Possible Effects of Cognitive Training on Sensory Inference

**Figure 2:**
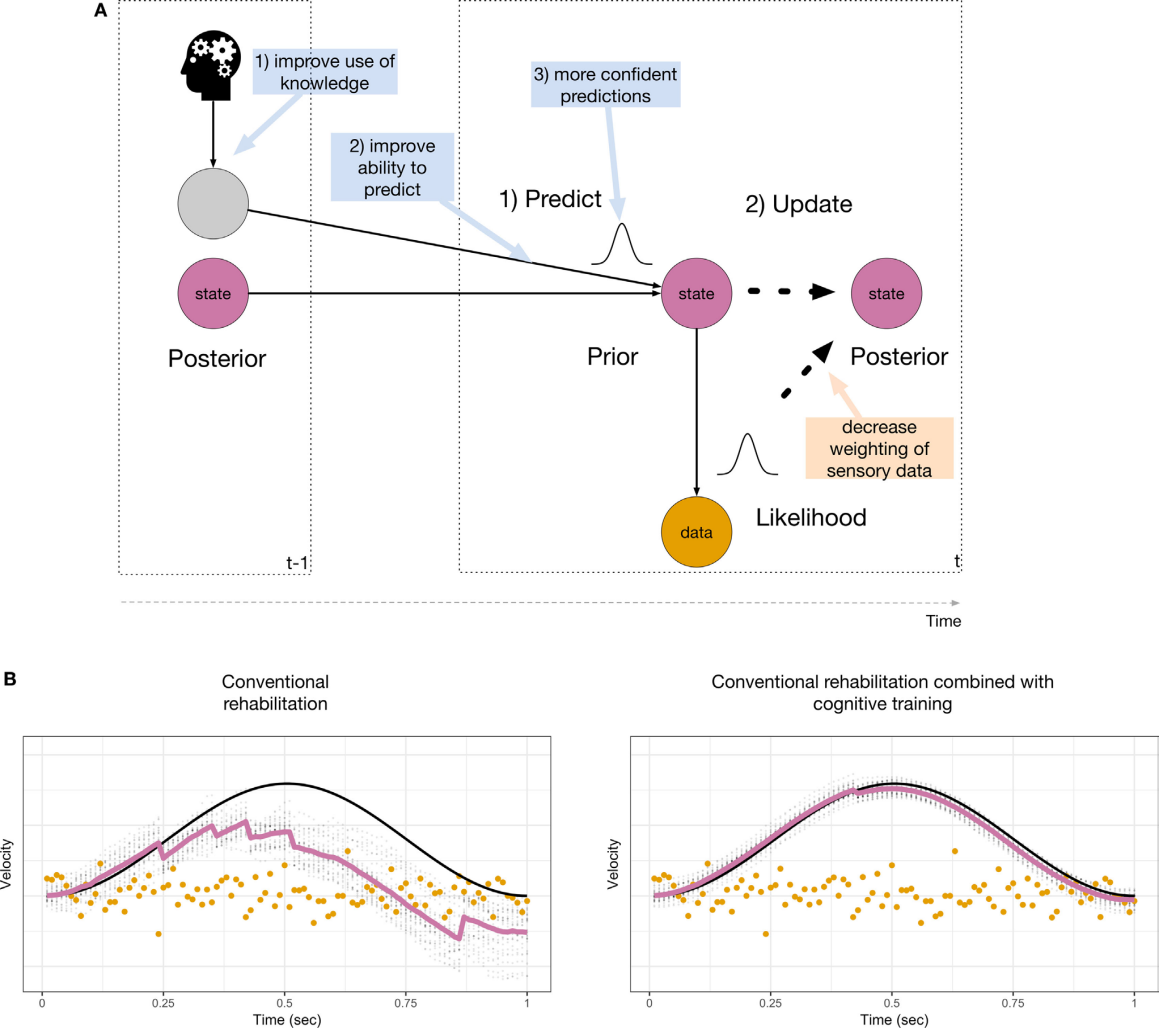
**(A)** Suggested interventions through both conventional rehabilitation (orange box) and cognitive training (blue boxes). Most conventional rehabilitation strategies are targeted at reducing the influence of the sensory data, whereas cognitive training targets the inclusion of prior knowledge for sensory inference. **(B)** Improvements that may be achieved by conventional rehabilitation (left) and additional cognitive training (right). Inference is improved when abnormal sensory data are ignored (compare with Figure 1D, bottom right).

In the context of the probabilistic model used for sensory inference, we can envisage distinct ways in which cognitive training may improve vestibular sensory inference. This is illustrated in Figure 2A (blue boxes). In the absence of sensory signals, any residual ability to detect head motion depends on patients’ ability to predict their head velocity. This prediction is equivalent to constructing a dynamic prior prediction of head velocity, and this requires the following steps: (1) knowledge about head movements from efference copies and cognitive sources is converted into an expected head velocity. (2) The head velocity is predicted. (3) The prediction of head velocity is made, but is uncertain. In order to improve inference, the confidence predictions are made should be increased. In Bayesian inference, this leads to a decreased weighting of the sensory data. This only leads to improved inference if an accurate process model is used. The process model used for sensory inference during active movement can be used off-line in the service of mental simulations, i.e., to imagine self-motion (34, 35). Indeed, there is evidence for the involvement of the vestibular system in spatial perspective taking tasks (50, 51). Although this has not previously been investigated, it is likely that the brain must simulate motion of the self in order to perform cognitive tasks. This ability may be reduced in BVP patients (6). We suggest that mental body rotation training may enable patients to improve their use of knowledge about the dynamics of head movements and to rehearse simulating head motion without the requirement of performing sensory inference. Cognitive training of head and body movements *via* mental imagery will help patients not only to improve their ability to predict movement and the ensuing sensory consequences, but also to increase their confidence in these predictions.

Figure 2B illustrates the results of simulating improvements due to conventional rehabilitation (left) and additional cognitive training (right). Conventional rehabilitation may enable BVP patients to improve their inference during a predictable movement when abnormal sensory data are down-weighted (compared with Figure 1D, bottom right). This is achieved by increasing the width of the sensor noise distribution. The estimated amplitude of the movement is still attenuated. Cognitive training may enable patients to improve their use of knowledge derived from their motor system, other sensory modalities (vision, proprioception) or memory and mental imagery to make better and more confident prior predictions of head movements. This is achieved by decreasing the width of the dynamic prior and yields greater improvements for sensory inference. In our model, the improvements due to cognitive training shown in Figure 2B (right) are akin to running the model in a simulation model.

During the course of rehabilitation, patients can learn to construct improved models of their head movements and use these for sensory inference. In addition, they can learn to rely more on their internal estimates and thereby increasingly ignore the abnormal sensory data from their vestibular end organs. More generally, mental imagery training, and other cognitive training methods, for example, targeting executive functions, may help reduce the cognitive load that BVP patients experience (10–12). In order to achieve the re-weighting of the prior predictions while simultaneously inhibiting sensory information, training of inhibition could help patients with BVP. Furthermore, a training of selective attention could lead to better allocation of attentional resources. It may also be beneficial to use cognitive training in combination with galvanic vestibular stimulation (52). Increasing the sensor noise could help patients to down-weight the abnormal sensory data. As a result, patients would be more responsive to cognitive training. Cognitive processes, such as imagined self-motion, have been shown to affect self-motion processing, a case in point being the study by Nigmatullina et al. (42). Even though our computational model was not conceived to make quantitative predictions, it is in line with their results. We are proposing that, in order to be effective, cognitive training methods should be designed with this computational framework in mind.

## Conclusion

The discussion about the consequences of BVP is largely dominated by focusing on the absence of vestibular sensory information and the use of other sensory sources, such as vision and proprioception. This has guided the conceptualization of treatments and rehabilitation. In patients with BVP, conventional treatments are often insufficient and there are other suitable entry points for interventions. Sensory processing involves prior knowledge about the world and this is necessary for correct inference of physical motion stimuli. Erroneous self-motion perception in BVP patients can be reduced by assigning more weight to prior knowledge and disregarding uninformative sensory data. Cognitive training is a promising tool to rebalance the mechanisms underlying sensory inference in order to react to the chronic loss of sensory data.

## Author Contributions

AE, CS, DV, MC, and FM wrote the manuscript. AE and FM planned and performed the simulations.

## Conflict of Interest Statement

The authors declare that the research was conducted in the absence of any commercial or financial relationships that could be construed as a potential conflict of interest.
